# G4LDB 3.0: a database for discovering and studying G-quadruplex and i-motif ligands

**DOI:** 10.1093/nar/gkae835

**Published:** 2024-09-25

**Authors:** Qian-Fan Yang, Xu-Rui Wang, Yu-Huan Wang, Xing-Hong Wu, Run-Yu Shi, Yan-Xi Wang, Hao-Ning Zhu, Shu Yang, Ya-Lin Tang, Feng Li

**Affiliations:** Key Laboratory of Green Chemistry and Technology of Ministry of Education, College of Chemistry, Sichuan University, No. 29, Wangjiang Road, Chengdu 610064, China; Key Laboratory of Green Chemistry and Technology of Ministry of Education, College of Chemistry, Sichuan University, No. 29, Wangjiang Road, Chengdu 610064, China; Key Laboratory of Green Chemistry and Technology of Ministry of Education, College of Chemistry, Sichuan University, No. 29, Wangjiang Road, Chengdu 610064, China; Key Laboratory of Green Chemistry and Technology of Ministry of Education, College of Chemistry, Sichuan University, No. 29, Wangjiang Road, Chengdu 610064, China; Key Laboratory of Green Chemistry and Technology of Ministry of Education, College of Chemistry, Sichuan University, No. 29, Wangjiang Road, Chengdu 610064, China; Key Laboratory of Green Chemistry and Technology of Ministry of Education, College of Chemistry, Sichuan University, No. 29, Wangjiang Road, Chengdu 610064, China; West China School of Pharmacy, Sichuan University, No. 17, Section 3, Southern Renmin Road, Chengdu 610041, China; West China School of Pharmacy, Sichuan University, No. 17, Section 3, Southern Renmin Road, Chengdu 610041, China; Beijing National Laboratory for Molecular Sciences (BNLMS), Center for Molecular Sciences, State Key Laboratory for Structural Chemistry of Unstable and Stable Species, Institute of Chemistry, Chinese Academy of Sciences, Zhongguancun North First Street 2, Beijing 100190, China; Key Laboratory of Green Chemistry and Technology of Ministry of Education, College of Chemistry, Sichuan University, No. 29, Wangjiang Road, Chengdu 610064, China

## Abstract

Non-canonical nucleic acid structures, such as G-quadruplex (G4) and i-Motif (iM), have garnered significant research interest because of their unique structural properties and biological activities. Thousands of small molecules targeting G4/iM structures have been developed for various chemical and biological applications. In response to the growing interest in G4-targeting ligands, we launched the first G4 Ligand Database (G4LDB) in 2013. Here, we introduce G4LDB 3.0 (http://www.g4ldb.com), an upgraded version featuring extensive enhancements in content and functionality. The new version includes over 4800 G4/iM ligands and approximately 51 000 activity entries. Key upgrades include advanced search capabilities, dynamic knowledge graphs, enhanced data visualization, along with a new dynamic analysis function that automatically displays ligand structure clustering results and chemical space distribution. With these updates, G4LDB 3.0 further evolves into a comprehensive resource and valuable research tool. The significant improvements address the increasing demand for efficient data handling and user experience, highlighting the critical role of G4LDB in advancing research on G-quadruplexes and i-motifs.

## Graphical Abstract

**Graphical Abstract:**
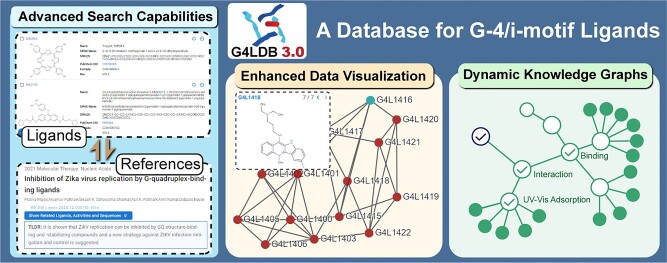

## Introduction

The past decade has witnessed the growing interest in biologically important non-canonical nucleic acid structures, such as G-quadruplex (G4) and i-Motif (iM), due to their unique structural features and potential biological activities (1–3). G4s, composed of stacked ‘G-quartets’, are prevalent in the human genome and are involved in various biological processes, including transcription (4), translation (5) and cell aging (6). These structures are also implicated in diseases (7–9), such as cancer, diabetes and neurodegenerative disorders. Similarly, iMs, which are cytosine-rich quadruplex structures, also play crucial roles in key genomic regions (10), including telomeres and proto-oncogene promoters.

Given their critical physiological functions, G4 and iM structures are prime targets for drug development. Numerous small molecules have been designed to recognize (11), regulate (12) and probe these structures in living cells (13–15) and *in vivo* (16). Traditional G4-targeting ligands, such as telomestatin and pyridostatin, have demonstrated substantial preclinical efficacy by stabilizing G-quadruplexes and disrupting key biological processes in cancer cells (17). Notably, several G4-targeting ligands, such as CX-5461 (18) and CX-3543 (19,20), have advanced to clinical trials. CX-5461, for instance, is in phase I/II trials for treating cancers with BRCA1/2 or HRD mutations and has shown promise in early studies (18).

In response to the growing interest in G4 ligands, we established one of the first G4 ligand databases, G4LDB, in 2013 (21). This database was initially designed to support research by collecting and organizing data solely on G4 ligands. An updated version, G4LDB 2.2, was released in 2021 (22), which not only recorded more G4 ligands and their activity entries but also included iM ligands as well as enhancements in the database structure and the user interface.

Here, we introduce G4LDB 3.0 (http://www.g4ldb.com), the latest version of our database, featuring significant upgrades in both content and functionality. This version includes over 4 800 G4 and iM ligands, and approximately 51 000 activity entries. Key upgrades in G4LDB 3.0 include advanced search capabilities using Elasticsearch (ES), dynamic knowledge graphs and enhanced data visualization techniques. The advanced search capabilities now allow for more precise and faster retrieval of information, expanding the scope of searchable content and improving the efficiency and accuracy of searches. Dynamic knowledge graphs provide interactive and visually engaging representations of ligand structures and their activities, facilitating quicker orientation and understanding of complex data relationships. Enhanced data visualization techniques offer improved ways to explore and interpret the vast amount of data, making it easier for researchers to derive meaningful insights. Additionally, a new dynamic analysis function has been added. With these advancements, G4LDB 3.0 has evolved into a comprehensive resource and invaluable tool for researchers, addressing the increasing demand for efficient data handling and user experience, and advancing the study of G-quadruplexes and i-motifs.

## Materials and methods

The methods employed for G4LDB 3.0 are consistent with those used in G4LDB 2.2, as previously described. Any new methods or significant modifications are detailed below.

### Database implementation

The G4LDB 3.0 employs a hybrid architecture. ES is utilized to manage unstructured literature data, while structured data, such as ligands and activities, are maintained in PostgreSQL. ES leverages an inverted index to expedite the retrieval of unstructured literature. During the ES indexing phase, automated techniques are employed to parse and store PDF content. The PyMuPDF (https://github.com/pymupdf/PyMuPDF) library reads PDFs and heuristic rules using regular expressions segment and merge the literature into sections. Each section forms a single ES document, with fundamental information about the literature and ligands attached as metadata.

During the retrieval phase, documents that best match the user’s search query are identified through ES’s inverted index mechanism. Different weights are assigned to various segments to enhance the retrieval of the most relevant content. To improve search efficiency, only the top-ranked documents are returned. Specifically, the server retains documents with a relevance score >60% of the highest score in that search.

### Retrieval strategy and search result presentation

The search results in G4LDB are generated using a combination of Structured Query Language (SQL)-based and ES-based searches. The SQL-based search matches the user’s query with specific fields in the PostgreSQL database, such as ligand names, to retrieve exact matches. These results are displayed as ‘Recommended Entries’, typically showing the top 1–3 entries most closely associated with the search term. The ES-based search examines the entire collection of unstructured literature to find any mention of the search term and compiles relevant studies and their associated ligands into the ‘Related Entries’ section.

### Knowledge graph construction

#### Ligand structure clustering

Ligand structures are clustered based on their structural Tanimoto similarity, calculated using the RDKit cheminformatics toolkit (version 2023_09_1b1, http://www.rdkit.org, DOI: 10.5281/zenodo.8413907). A threshold of 0.7 is used to define clusters.

#### Graph visualization

Cytoscape.js (23) is employed as the visualization engine, with the Euler algorithm used for graph layout. The Cascading Style Sheet (CSS) interface provided by Cytoscape.js is utilized to manage graph styles.

#### Ligand structure clustering map

This map is an undirected graph where each vertex’s attributes are retrieved from the G4LDB relational database. Heuristic rules are applied to combine multiple attributes of the same vertex.

#### Ligand activity hierarchical map

This map is a directed acyclic graph, dynamically constructed from the relational database. Ligands serve as root nodes, with activities forming the paths and leaf vertices.

#### Family display in analysis page

Each family contains multiple clusters, and each cluster is a connected graph. A virtual graph node is created for all clusters under a family. Nodes from the same cluster are assigned a common parent attribute, pointing to the virtual cluster node. The Fcose (24) algorithm is used for the layout, with the virtual cluster node drawn as a constraint box.

#### Attribute legend

All attribute sets are collected from the graph. Unique attribute values are computed to form sets, and their counts are tallied. The names are quickly sorted alphabetically. Colors are assigned to the set by proportionally adjusting the lightness in the HSL (Hue, Saturation and Lightness) color space based on the original data. Legend items are then drawn sequentially according to the order in the set.

### Short summary for literatures

Short summaries, termed TLDR (short for ‘Too Long, Didn’t Read’) (https://www.semanticscholar.org/product/tldr), for all literatures are automatically generated by Semantic Scholar, providing a concise single-sentence summary of each document.

## Results

### Overview of G4LDB 3.0

G4LDB 3.0 is the latest and most advanced version of the quadruplex nucleic acid ligand database, designed to provide an extensive collection of small molecular ligands for G4 and iM structures. This version not only expands on the database’s content but also significantly enhances its functionality to support cutting-edge research in this field.

Building on the foundation of previous versions (21,22), G4LDB 3.0 now boasts 4 851 G4 and iM ligands, marking a substantial increase in both quantity and diversity (Table 1). Specifically, the database contains 4 563 G4 ligands and 288 iM ligands. Ligands are categorized based on their binding type, with those labeled as ‘G4L’ classified as G4 binders and those starting with ‘iML’ categorized as i-motif binders. The number of activity entries has also grown dramatically to 51 024, with 48 982 activities related to G4 ligands and 2 042 activities related to iM ligands. This growth reflects the rapid development and increasing interest in G4 and iM ligands in recent years, highlighting their expanding roles in scientific research and drug development.

**Table 1.** Overview of data across different versions of G4LDB

		G4LDB 1.0	G4LDB 2.2	G4LDB 3.0
Data capacity	G4 ligands	1 105	3 099	4 563
	G4 ligand activities	4 751	27 807	48 982
	iM ligands	0	110	288
	iM ligand activities	0	883	2 042
Activity statistics	Molecular interaction	1 955	18 595	34 298
	Biological activity at the molecular level	1 105	1 642	3 858
	Biological activity at the cellular level	1 691	8 096	12 144
	Biological activity at the *in vivo* level	0	357	724

Key improvements in G4LDB 3.0 include:

- **Advanced search capabilities**: Utilizing ES, the database now supports more precise and faster information retrieval, enhancing the scope and efficiency of searches.
- **Dynamic knowledge graphs**: These provide interactive, visually engaging representations of ligand structures and their activities, facilitating a deeper understanding of complex data relationships.
- **Enhanced** **data visualization techniques**: These improvements offer better methods for exploring and interpreting vast amounts of data, aiding researchers in deriving meaningful insights.

In addition to these enhancements, G4LDB 3.0 features a new dynamic analysis function, allowing users to display and analyze ligand structure clustering results and chemical space distribution. These advancements make G4LDB 3.0 a comprehensive and invaluable resource for researchers, addressing the increasing demand for efficient data handling and user experience, and advancing the study of G-quadruplexes and i-motifs.

## New features

### User interface Improvements

**Figure 1.**
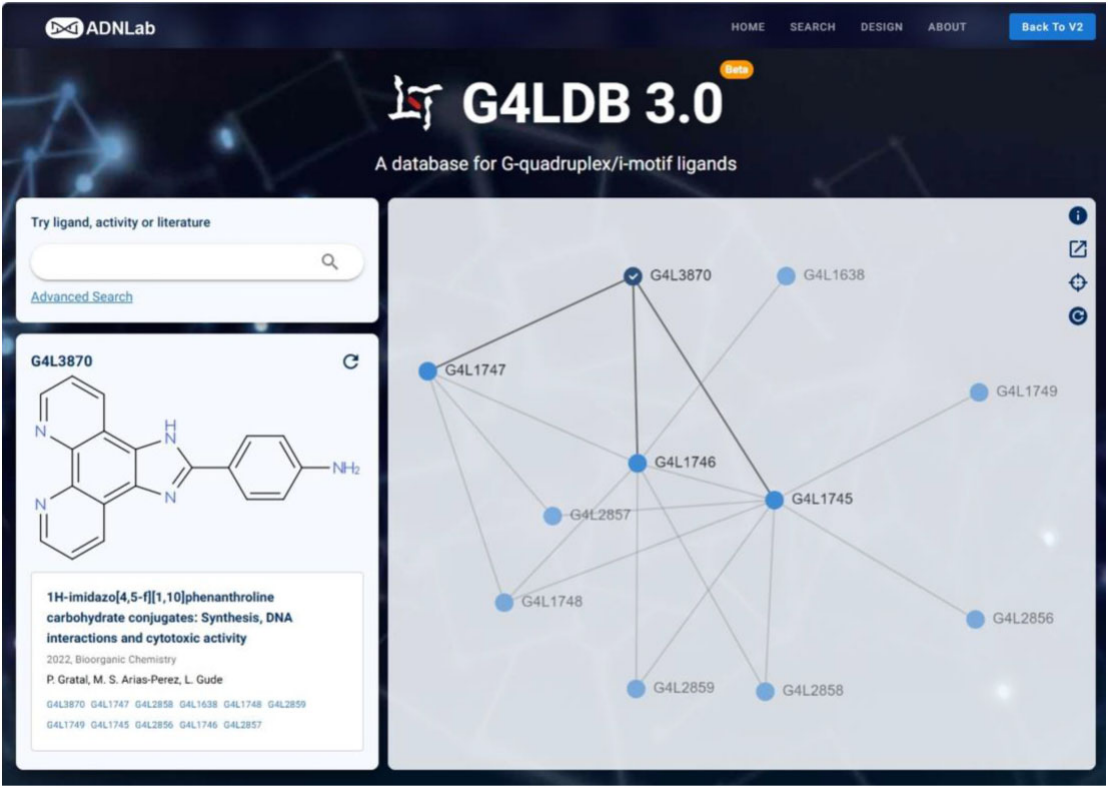
The main page of G4LDB 3.0.

Several pages have been redesigned in G4LDB 3.0, including the main page of the website, to enhance user engagement and usability. In the latest version, the main page now randomly displays a newly included ligand (Figure 1), showcasing its molecular formula, reported literature, journal and authors. This feature provides users with fresh and relevant content each time they visit. Additionally, an interactive Ligand Structure Clustering Map of the selected ligand is also presented. This dynamic map encourages users to explore related ligands and their structural similarities, fostering a more engaging and exploratory experience.

### Enhanced search capabilities

**Figure 2.**
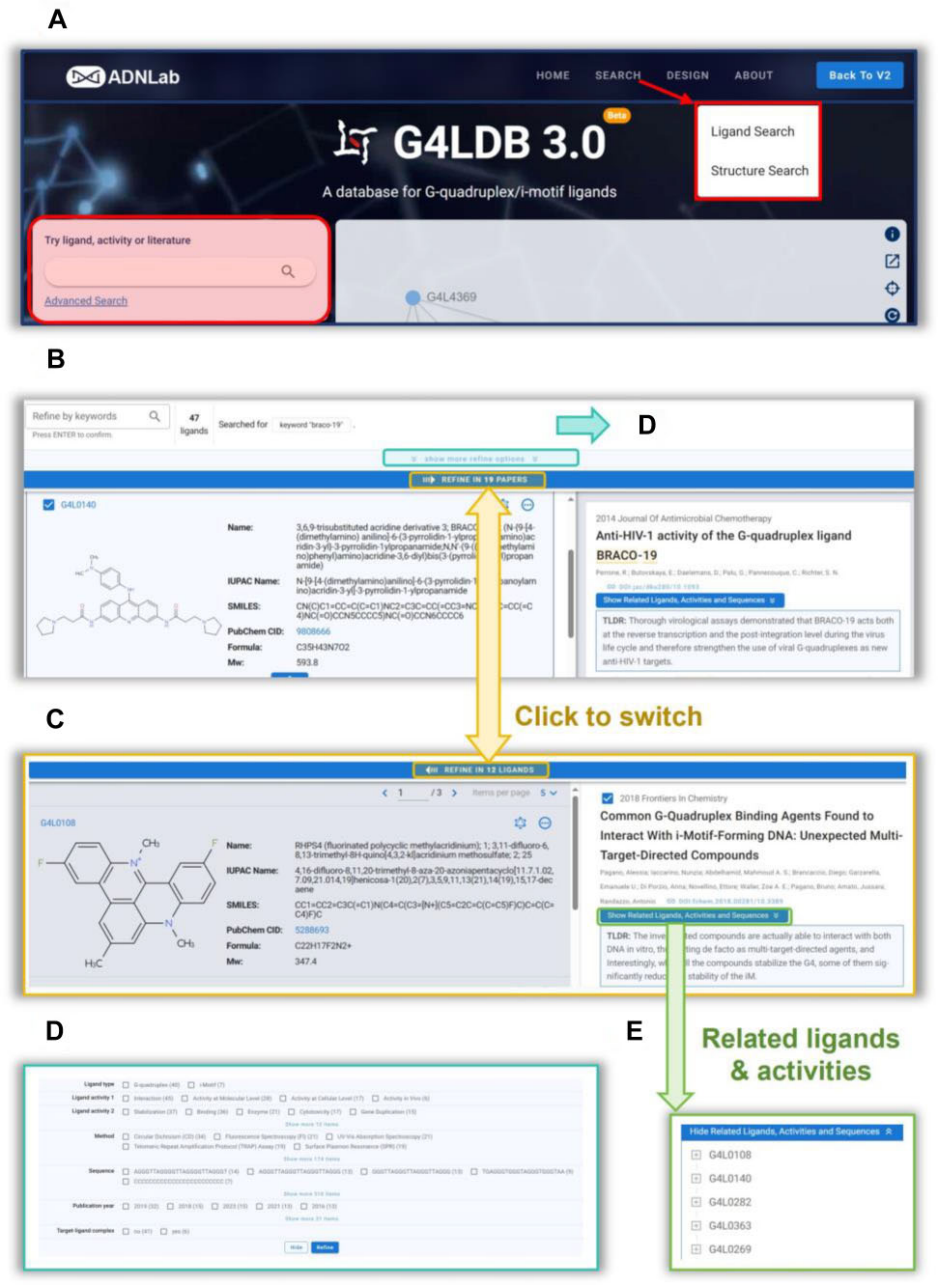
Enhanced search capabilities of G4LDB 3.0. (**A**) Various search entries on the main page. (**B**) The redesigned ’Search Result’ page with a split-window design, showing the ***Ligand Pane*** activated. (**C**) The redesigned ‘Search Result’ page with the ***Paper Pane*** activated. (**D**) The refine pane. (**E**) The expandable table that consolidates ligands, related activities and nucleic acid sequence information mentioned in the related paper.

The search functionality has been significantly redesigned in G4LDB 3.0 to better meet user needs, retaining key features from G4LDB 2.2, such as the simple search box on the main page and advanced search options, including ‘Ligand Search’ and ‘Structure Search’ (Figure 2A).

The new designed ***Search Result*** page features a split-window design, dividing the screen into ***Ligand Pane*** (left) and ***Paper Pane*** (right). Users can switch between the panes to activate either the ligand or literature side for operations. When item(s) are selected from the table in one pane, users can refine the content displayed in the other pane, creating a more interactive and dynamic experience (Figure 2B and C). All refine functionalities are integrated at the top of the page for ease of access (Figure 2D).

To provide a comprehensive view of related literature, the newly added ***PaperPane*** includes detailed information such as titles, authors, journals and DOI links. To assist users in evaluating the relevance of papers, the paper pane features automatically generated short summaries (termed TLDR) powered by SemanticScholar. These summaries provide a quick overview of the paper’s content, enabling users to determine its relevance more efficiently. Additionally, the ES engine automatically highlights relevant content within the literature, helping users swiftly pinpoint information pertinent to their research queries.

Each paper also includes an expandable table that consolidates ligands, related activities and nucleic acid sequence information mentioned in the paper (Figure 2E). This table allows users to explore related or interesting ligands more conveniently, promoting a deeper and more interconnected understanding of the data.

With the integration of ES into our database, users can now obtain a more comprehensive and relevant set of search results. For instance, a search for BRACO-19, a well-known quadruplex binder, yielded 47 hits (Figure 2B), which are categorized into distinct classes to enhance information retrieval efficiency and relevance. The results are divided into several main categories:

- **Direct entries for BRACO-19:** This includes two entries specifically for BRACO-19 itself (G4L0140 and iML0028), which are listed under the ‘Recommended Entries’ section. This allows users to quickly access detailed data about this ligand.
- **Comparative studies:** This category encompasses 43 entries where BRACO-19 is referenced in different study contexts and is listed under the ‘Related Entries’ section:
  - 38 entries involve related ligands used as positive controls in studies, where BRACO-19 was employed to demonstrate similar activities.
  - Two entries feature related ligands used in comparative studies, where BRACO-19 serves as a benchmark to show different activities.
  - Three entries are discussed in studies comparing both the similarities and differences in activity with BRACO-19.
- **Competitive binding experiments: This includes two entries where BRACO-19 is utilized in competitive binding assays**.

The enhanced search engine enables users to filter and view information more effectively, providing both direct and extended insights into BRACO-19 and related studies.

The operational logic of the database remains consistent with previous versions, ensuring a familiar user experience. To further assist users, we have included detailed user instructions in the Electronic Supplementary Information, providing step-by-step guidance on key functionalities, including navigating to the ligand detail page (Supplementary Figures S1 and S2), browsing and refining specific activity data for a ligand (Supplementary Figure S3) and accessing ligand complex information (Supplementary Figure S4). Due to the new retrieval logic, the browse functionality from the old version is no longer available in the new interface. However, users can switch back to G4LDB 2.2 through the navigation bar whenever they need to access the browse function.

### Dynamic knowledge graphs

**Figure 3.**
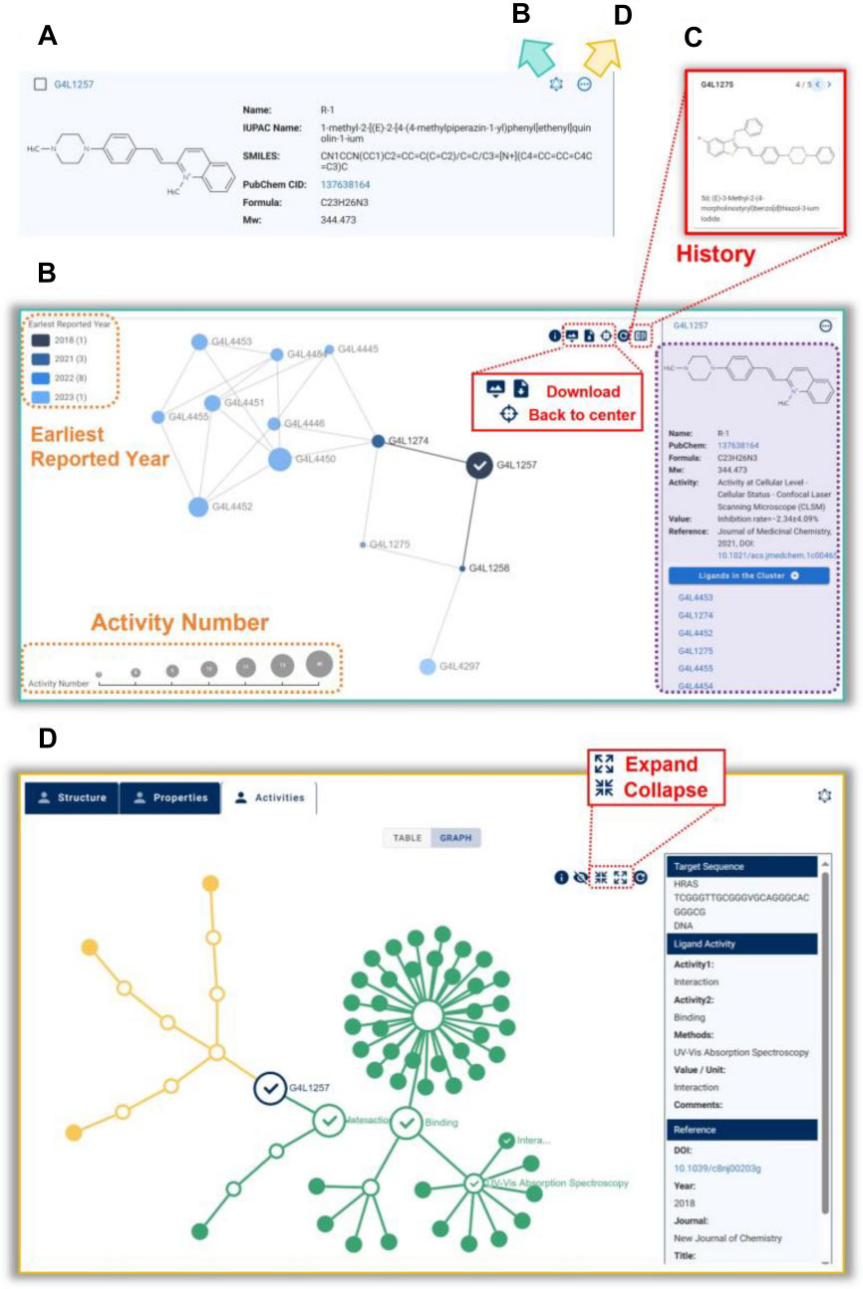
Dynamic knowledge graphs of G4LDB 3.0. (**A**) All graphs are accessible directly from the ligand pane list. (**B**) A ***Ligand Structure Clustering Map*** of the ligand G4L1257. (**C**) The molecular structure and name of a previously clicked ligand accessed via the ***History*** icon. (**D**) A ***Ligand Activity Hierarchical Map*** of the ligand G4L1257.

To meet the diverse needs of users, we have embedded multiple visual dynamic knowledge graphs in the new version, accessible directly from the ligand pane list (Figure 3A). These graphs aim to facilitate the exploration and analysis of ligand structures and activities, providing a more intuitive and interactive user experience.

We have implemented two main types of dynamic knowledge graphs: Ligand Structure Clustering Map and Ligand Activity Hierarchical Map.

In the ***Ligand Structure Clustering Map***, ligands with structural similarities are displayed as a dynamic network (Figure 3B). Each node represents a ligand, and nodes are connected if their structural similarity exceeds 0.7. The size and color of each node indicate the number of activities and the year the ligand was first reported, respectively. Detailed information about the selected ligand is automatically displayed on the right side, along with a list of all ligands shown on the page. The top right corner features a series of icons for customizing and manipulating the network, including a ***History*** icon (toggle counter) that allows users to trace back and view the molecular structures and names of previously clicked ligands (Figure 3C).

In the ***Ligand Activity Hierarchical Map***, all activities of a ligand are grouped and visualized (Figure 3D). Users can expand or collapse each level along the hierarchy of Activity 1, Activity 2, Method and Value. Clicking on a value node reveals all related information on the right side, including nucleic acid sequences, activities and literature details. This page also includes icons for network customization and manipulation at the top right corner.

### Analysis function

**Figure 4.**
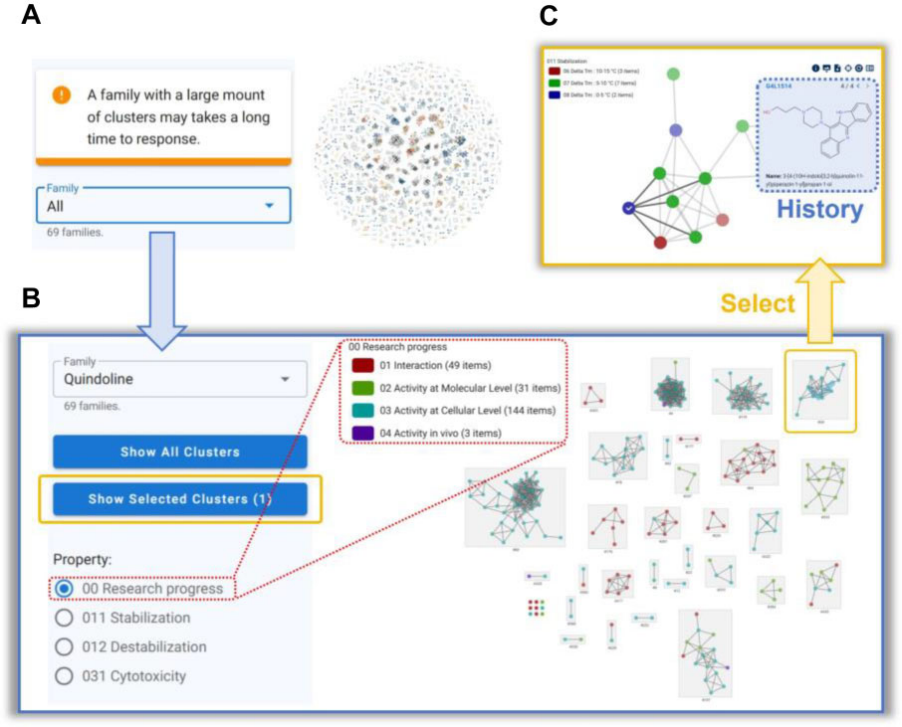
New analysis page in G4LDB 3.0. (**A**) The main page where all ligands are categorized into 69 families. (**B**) A ***Ligand Structure Clustering Map*** of the quindoline family, displaying ligands with colors assigned according to the selected property attributes. (**C**) Focused view of the selected cluster and detailed information about the selected ligand.

To better present the structure–activity relationship of G4 ligands, we have designed a new ‘Analysis’ page (Figure 4A). On this page, all ligands included in the database are categorized into 69 families based on their core molecular frames. Users can select any family to view its ***Ligand Structure Clustering Map*** (Figure 4B), where each node represents a ligand. The nodes are color coded based on corresponding property attributes. We have already analyzed the research progress and several key quantitative activities of G4 ligands, including their stabilization/destabilization abilities and cytotoxicities. To make the visualization more intuitive, we have transformed the raw quantitative activities into semi-quantitative categories for color assignment. Additionally, we will continue to add more quantitative and qualitative activity analysis data soon.

By clicking on any cluster in the map (use Ctrl + left mouse button for multiple selections) and then clicking ’show selected clusters’ on the left, users can further focus on the selected cluster(s) (Figure 4C). Clicking on any ligand will display detailed information about that ligand. This page also includes icons for network customization and manipulation, as well as a ***History*** icon, located at the top right corner.

## Supplementary Material

gkae835_Supplemental_File

## Data Availability

The database is now publicly accessible through the URL http://www.g4ldb.com.
