# Supplementary material for: G4LDB 3.0: a database for discovering and studying G-quadruplex and i-motif ligands
Source: Nucleic Acids Res. 2024 Sep 25;53(D1):D91–8. doi: 10.1093/nar/gkae835 (PMC11701546; doi:10.1093/nar/gkae835)
Supplement: gkae835_Supplemental_File [file gkae835_supplemental_file.docx]

**Supporting Information**

**G4LDB 3.0: a database for discovering and studying G-quadruplex and i-Motif ligands**

**AUTHORS**

Qian-Fan Yang^1†^, Xu-Rui Wang^1†^, Yu-Huan Wang^1^, Xing-Hong Wu^1^, Run-Yu Shi^1^, Yan-Xi Wang^1^, Hao-Ning Zhu^2^, Shu Yang^2^*, Ya-Lin Tang^3^* and Feng Li^1^*

^1^ Key Laboratory of Green Chemistry and Technology of Ministry of Education, College of Chemistry, Sichuan University, Chengdu, 610064, China

^2^ West China School of Pharmacy, Sichuan University, Chengdu, 610041, China

^3^ Beijing National Laboratory for Molecular Sciences (BNLMS), Center for Molecular Sciences, State Key Laboratory for Structural Chemistry of Unstable and Stable Species, Institute of Chemistry, Chinese Academy of Sciences, Beijing 100190, China

† Joint Authors: Qian-Fan Yang and Xu-Rui Wang.

* To whom correspondence should be addressed. Tel: +86-15881094081; Email: [windtalker_1205@scu.edu.cn](mailto:windtalker_1205@scu.edu.cn).

Correspondence may also be addressed to Ya-Lin Tang (Email: [tangyl@iccas.ac.cn](mailto:tangyl@iccas.ac.cn)) and Shu Yang (Email: [yangshu1106@scu.edu.cn](mailto:yangshu1106@scu.edu.cn)).

- **Navigating to the Ligand Detail Page**

1. On the Main Page (Figure S1), enter your search term in the search bar and click the search icon. This will take you to the Search Results Page.
2. On the Search Results Page, click on the ligand ID of any compound you are interested in (Figure S2, top). This will direct you to the Ligand Detail Page (Figure S2, bottom), where you can find detailed information about the selected ligand.


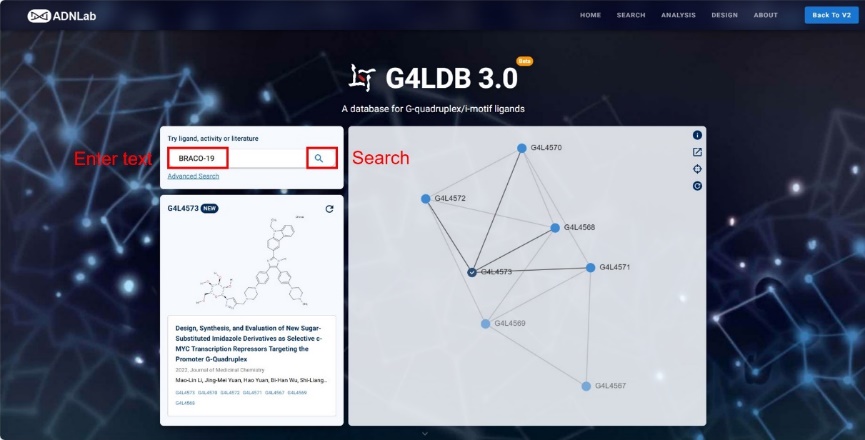


Figure S1. The main page of G4LDB 3.0, showing the search function for ligands, activities, and literature.


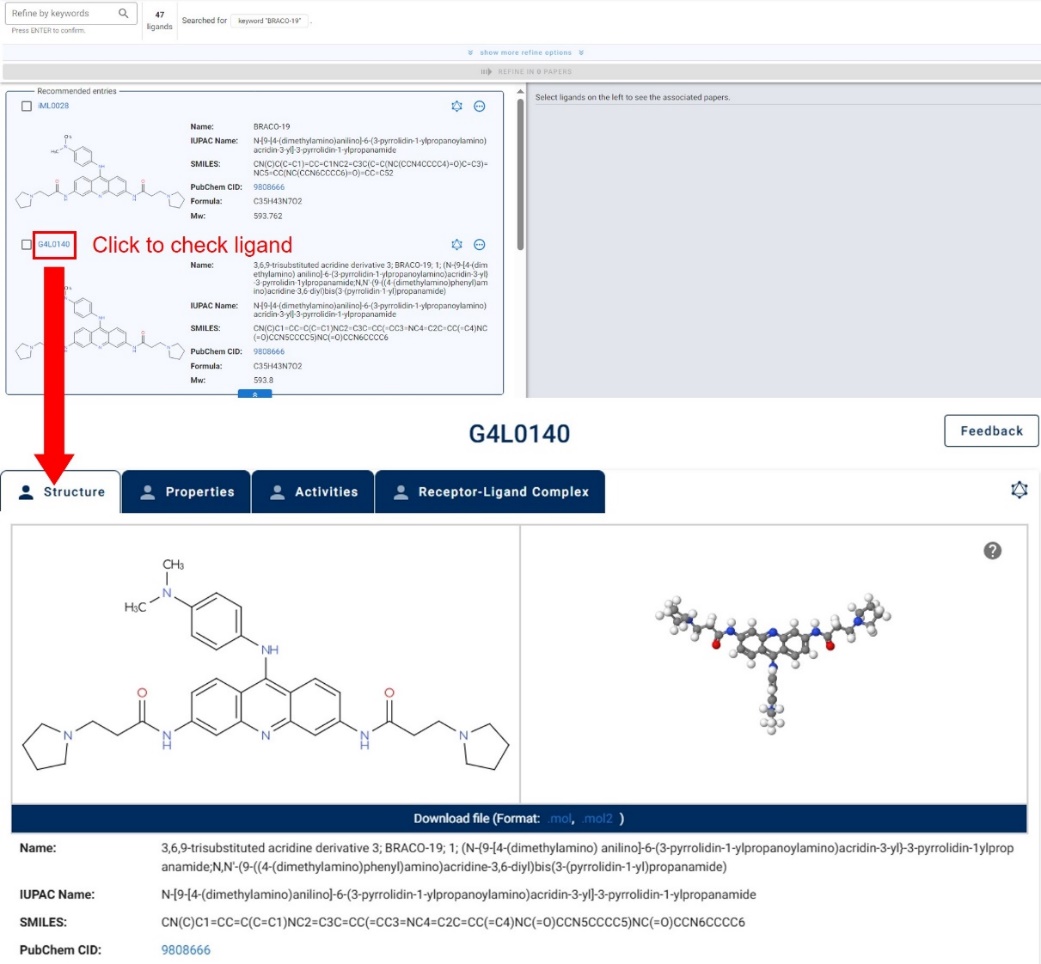


Figure S2. The Search Results Page (top) of G4LDB 3.0 showing search results for ligand G4L0140, and the Ligand Detail Page (bottom) displaying detailed information for the selected ligand G4L0140.

- **Browsing and Refining Specific Activity Data for a Ligand**

1. On the Ligand Detail Page, select the "Activities" tab to view activity data associated with the ligand.
2. Use the refine options (Figure S3, top) to filter specific activity types or to narrow down by the source of activity data, including publication year and journal.
3. Scroll down to see the filtered list of activities. Click on any row to expand and view detailed information in the data table (Figure S3, bottom).


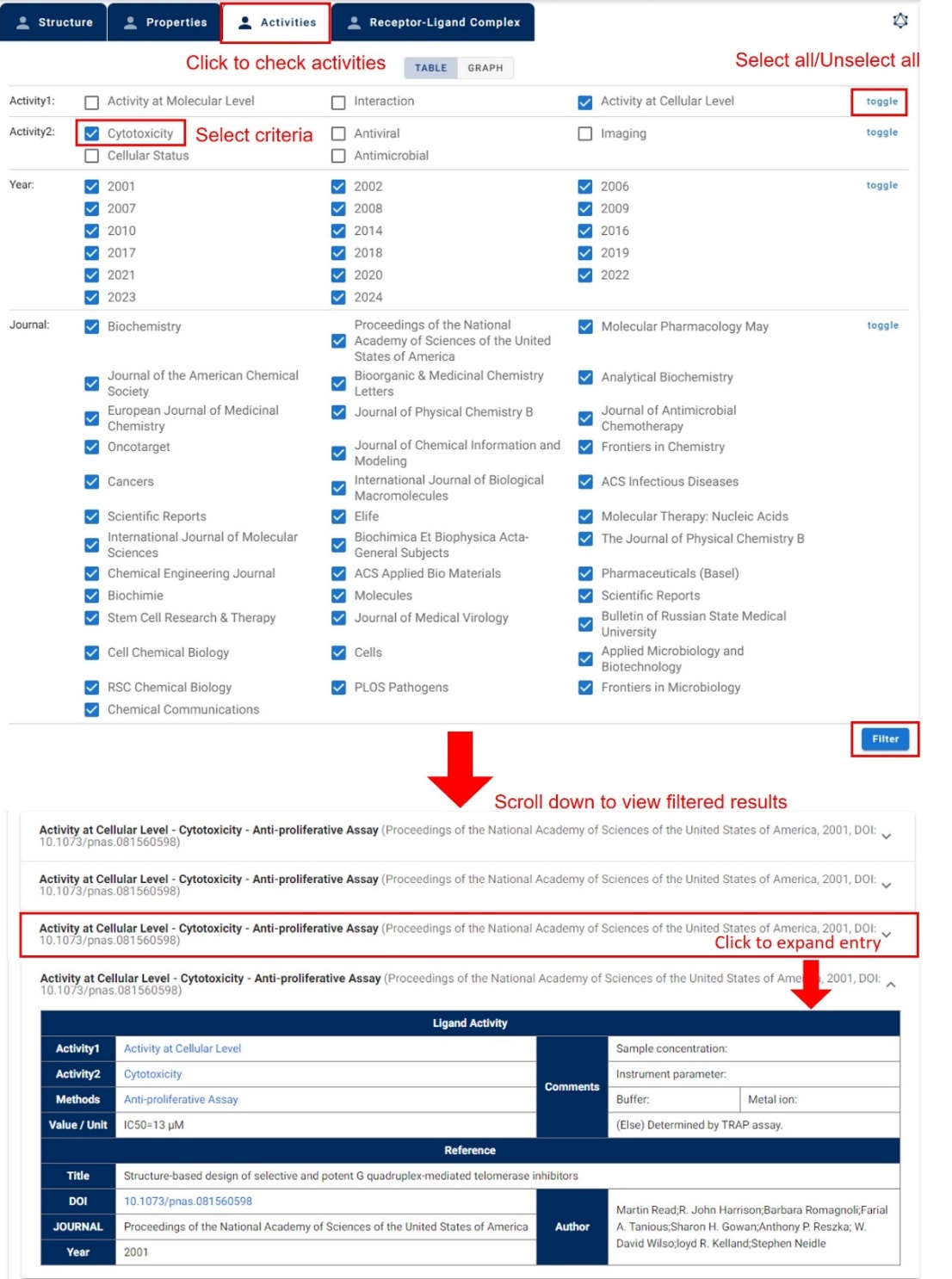


Figure S3. Example of browsing and refining specific activity data for ligand G4L0140. The top image shows the detailed refine options available under the "Activities" tab, while the bottom image displays a portion of the activity list and an expanded data table for one entry.

- **Accessing Ligand Complex Information (If Available)**

1. On the Ligand Detail Page, select the "Receptor-Ligand Complex" tab (Figure S4). This tab will only appear if there is complex data available for the ligand.
2. The bottom section of the page displays a list of available complexes (a ligand may have multiple complexes with various G4 sequences). Click on any row to expand and view detailed information, including PDB ID, source publication details, the sequence of the nucleic acid receptor, release date, and the method used to resolve the complex, such as X-ray diffraction or NMR.
3. For each complex, users can easily click the Docking button on the right side to access the docking page based on that specific complex.


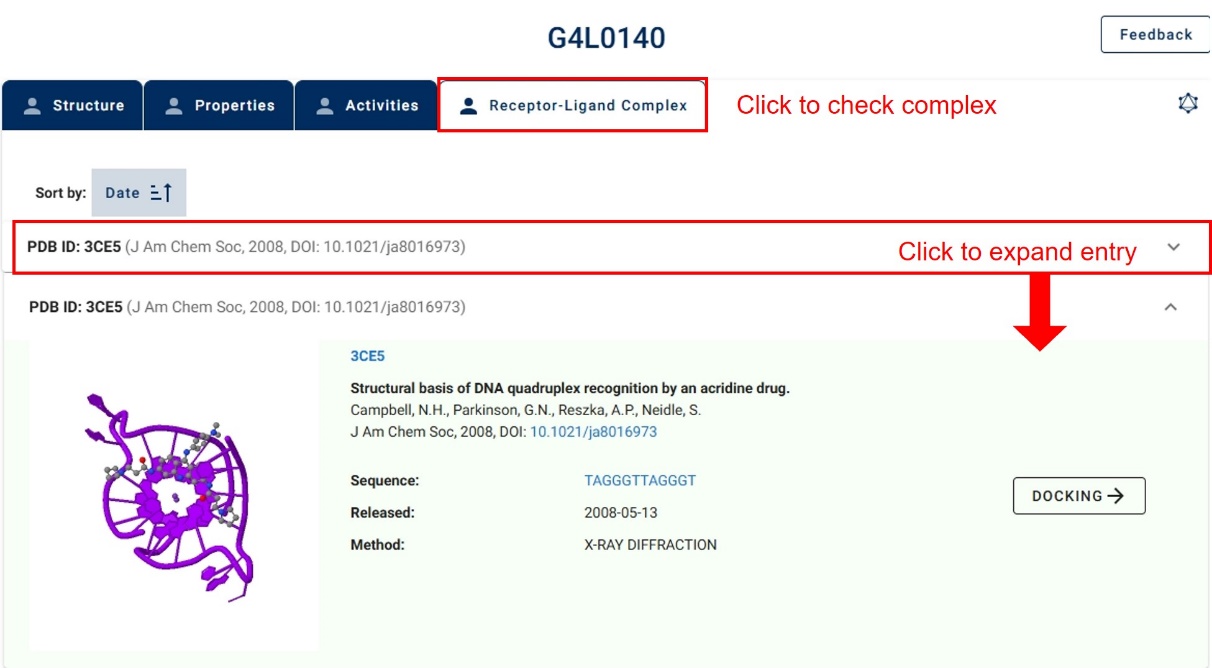


Figure S4. Example of the "Receptor-Ligand Complex" tab for ligand G4L0140, displaying a list of available complexes and expanded detailed information for one complex 3CE5.
